# Night Blindness in Cystic Fibrosis: The Key Role of Vitamin A in the Digestive System

**DOI:** 10.3390/nu11081876

**Published:** 2019-08-13

**Authors:** Lorenzo Norsa, Laura Zazzeron, Marialaura Cuomo, Laura Claut, Anna Marta Clotilde Bulfamante, Arianna Biffi, Carla Colombo

**Affiliations:** 1 Pediatric Hepatology Gastroenterology and Transplantation, ASST Papa Giovanni XXIII, 24027 Bergamo, Italy; 2 Cystic Fibrosis Center, Fondazione IRCCS Ca’ Granda Ospedale Maggiore Policlinico, University of Milan, 20121 Milan, Italy; 3 Department of Pediatrics, Fondazione MBBM Onlus/Ospedale San Gerardo, University of Milano-Bicocca, 20121 Milan, Italy; 4 Dipartimento di Fisiopatologia Medico-Chirurgica e dei Trapianti, Università degli Studi di Milano, 20121 Milan, Italy

**Keywords:** cystic fibrosis, vitamin A deficiency, short bowel syndrome

## Abstract

Vitamin A is a fundamental micronutrient that regulates various cellular patterns. Vitamin A deficiency (VAT) is a worldwide problem and the primary cause of nocturnal blindness especially in low income countries. Cystic fibrosis (CF) is a known risk factor of VAD because of liposoluble vitamin malabsorption due to pancreatic insufficiency. We describe a case of a 9-year-old girl who experienced recurrent episodes of nocturnal blindness due to profound VAD. This little girl is paradigmatic for the explanation of the key role of the gut–liver axis in vitamin A metabolism. She presents with meconium ileus at birth, requiring intestinal resection that led to a transient intestinal failure with parenteral nutrition need. In addition, she suffered from cholestatic liver disease due to CF and intestinal failure-associated liver disease. The interaction of pancreatic function, intestinal absorption and liver storage is fundamental for the correct metabolism of vitamin A.

## 1. Introduction

Humans, and in general higher animals, must obtain vitamin A from their diet, either as a pre-formed vitamin (retinol) from meat, eggs and milk, or as pro-vitamin carotenoids, primarily found in yellow fruit, vegetables and carrots [1].

These are both fat soluble molecules that need bile acid micelle solubilization to be digested. In the lumen of the small intestine, long chain fatty acid retinol esters (the most represented pre-formed vitamin in the diet) are hydrolyzed by pancreatic lipase and by enzymes on the brush border, and therefore free retinol can be absorbed by enterocytes. Beta carotene, the most abundant of carotenoids in the diet, does not require digestion to enter intestinal cells and diffuses through the cell membrane via facilitated transport linked to a surface receptor. In the cell, retinol is esterified again to retinyl ester (mainly with palmitate) and is embedded in chylomicrons together with beta carotene, other carotenoids and dietary lipids to be secreted in lymphatic vessels. Chylomicrons undergo remodeling processes resulting in chylomicron remnants, which are richer in retinyl esters and are up taken by the liver; here, retinol can be stored or can be bound to serum retinol binding protein (RBP4), a protein of hepatic origin, and delivered to peripheral tissues [2].

Normally the liver contains a two-year store of vitamin A, mainly as retinyl palmitate and stearate which can be hydrolyzed to retinol and secreted when needed [3].

Differently, carotenoids in the bloodstream are bound to high-density lipoprotein (HDL) and low-density lipoprotein (LDL) with other lipids [1].

These micronutrient compounds are either individually or collectively required throughout life and regulate many processes including cell differentiation, proliferation and development, embryogenesis, growth, normal metabolism and immune function. Moreover, beta carotene also has an important role as an antioxidant too. In retinal pigment epithelium retinol is efficiently up taken by a membrane receptor, stimulated by retinoic acid 6 (STRA6), with high affinity for RBP4, and while bound to a cellular RBP, is converted into 11-cis retinaldehyde, a chromophore that joins with the protein opsin to form rhodopsin, the molecular complex involved in vision [4].

Vitamin A deficiency (VAD) can be defined as serum retinol concentration lower than 0.70 μmol/L (less than 0.20 mg/L) [5] and is especially prevalent in low income countries [6], where malnutrition frequently occurs. Nevertheless, hypovitaminosis A due to inadequate intake is the leading cause of childhood blindness worldwide; while rare in developed countries [7], it can arise due to intestinal lipid malabsorption, pancreatic insufficiency, liver and bowel disease or surgical shortening.

According to these premises, we describe the case of a 9-year-old girl with a neonatal diagnosis of cystic fibrosis (CF) with cholestatic liver disease and a history of bowel surgical shortening presenting with night blindness secondary to VAD.

Data were collected retrospectively and anonymized after parents’ approval with informed consent signature. No ethical clearance was applicable according to national regulations.

## 2. Case Report

A full-term newborn girl developed abdominal distention with emesis and failure to pass meconium 12 h after birth. The pregnancy had been complicated by an antenatal diagnosis of polyhydramnios and dilated bowel loops. The mother was in good clinical condition without any history of vitamin deficit before or during pregnancy.

The baby underwent an extensive enterectomy with small bowel resection (involving 30 cm of the proximal ileum, the terminal atresic ileum, the ileocecal valve and the cecum) and ileostomy formation on day one of life.

The diagnosis of CF was confirmed by DNA analysis, showing compound heterozygosity for known disease-causing mutations (F508del/L1077del).

During the first months of life, the patient presented with abnormal liver enzyme levels with normal total and direct bilirubin levels. Abdominal ultrasound revealed mild hepatomegaly and steatosis.

Despite a severe intestinal malabsorption with steatorrhea (steatocrit of about 20%), both weight and height increased, and growth pattern was satisfactory.

Minimal enteral feeds were tolerated, and the baby was dependent on parenteral nutrition (PN) including fat-soluble vitamin supplementation (A 1300 UI/die, D 240 UI, E 4.2 UI, K 120 μg per day). According to CF nutritional guidelines [4], she received oral nutrition support and multivitamin supplementation (including vitamin A at 4000 UI daily).

The patient remained under close clinical follow-up until six months of age, when she developed a bowel obstruction due to adhesions, which required additional bowel surgery. After surgery, a total length of 102 cm of small bowel was measured with a termino-terminal ileocolic anastomosis without ileocecal valve.

For this condition of type 2 short bowel with intestinal failure, she was trained for home parenteral nutrition and discharged. Home PN (HPN) was successfully administered for the next 15 months when it was gradually stopped because of normal growth.

She was then regularly followed-up at the CF center with adequate oral dietary intake via oral nutrition, pancreatic enzyme replacement therapy and liposoluble vitamin supplementation at high dose.

At four years of age, she started complaining of slow dark adaptation and nyctalopia. No other symptoms were reported. An ophthalmic examination was performed: slit lamp examination was remarkable for a Bitot’s spot without retinal dystrophy; electroretinography (ERG) was within normal limits.

Night blindness due to systemic VAD was suspected and confirmed by a serum level of 0 mg/L (normal range of 0.30–0.70 mg/L), measured by high performance liquid chromatography (HPLC).

Fasting hypoglycemia was also documented, suggesting impaired intestinal absorption; thus, despite being within normal growth parameters, complementary HPN was restarted in order to provide additional calories and micronutrients.

Within the first month of HPN, the night blindness resolved.

**Figure 1:**
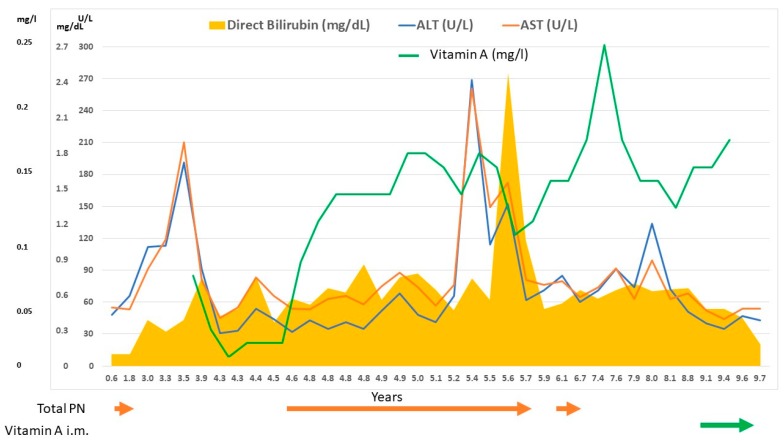
Correlation between liver function tests (AST—ALT—direct bilirubin), vitamin A levels, parenteral nutrition (PN) administration and vitamin A supplementation.

After 10 months of parenteral supplementation, despite treatment with ursodeoxycholic acid and efforts to optimize enteral feeding and cycling of HPN, severe intrahepatic cholestasis developed (Figure 1).

Therefore, in consideration of her optimal clinical conditions and normal levels of micronutrients, HPN was stopped again.

**Figure 2:**
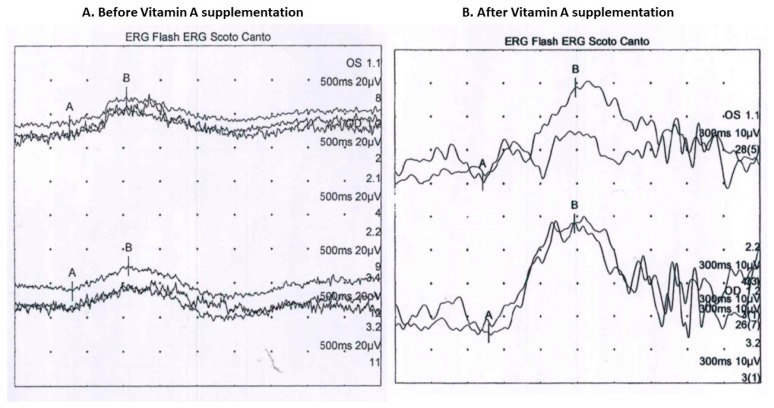
Patient’s full-field electroretinogram (ERG) (**A**) before vitamin A supplementation: decreased scotopic responses in both eyes and depressed photopic response in the left eye; (**B**) after vitamin A supplementation: normal.

Two years after HPN interruption, despite normal growth and no clinical symptoms, the patient’s ERG showed a significant decrease in scotopic responses in both eyes with a mild reduction in photopic responses in the left eye (Figure 2).

Serum vitamin A level was below 0.11 mg/L, while serum levels of the other liposoluble vitamins values were within normal range. Therefore, intramuscular vitamin A supplementation (100,000 UI monthly) was started and maintained, as serum vitamin A had remained only at trace levels; however, a prompt improvement in the ERG waveforms was observed.

## 3. Discussion

We describe for the first time the case of a child with short bowel syndrome associated with CF suffering from recurrent night blindness.

**Figure 3:**
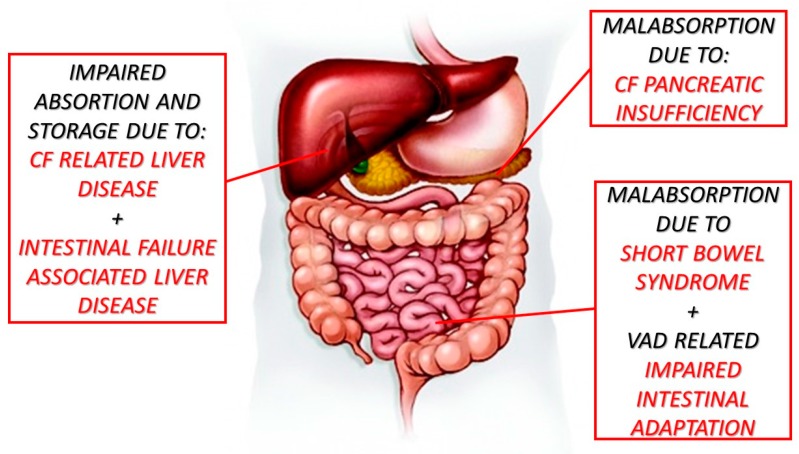
Risk factors related to vitamin A deficiency. CF = cystic fibrosis; VAD = vitamin A deficiency.

The patient described here is paradigmatic as all main risk factors for VAD were present due to an impaired gut–liver axis related to CF, meconium ileus which led to short bowel syndrome with absence of the terminal ileum and ileocecal valve, pancreatic insufficiency and cholestatic liver disease (Figure 3).

Bitot’s spots in combination with nyctalopia should promptly suggest the presence of acquired vitamin A deficiency.

First of all, vitamin A is necessary for the maintenance of the ocular surface, as it is involved in epithelial cell growth, regeneration, conjunctival epithelial cell RNA and glycoprotein synthesis helping to maintain the conjunctival mucosa and corneal stroma. Secondly, in the retina, retinol acts as the backbone of the photosensitive visual pigment in both rod and cone photoreceptors. The rod system is much more sensitive to vitamin A deficiency than the cone system. Therefore, nyctalopia is the most common and earliest symptom of vitamin A deficiency, whereas Bitot’s spot and conjunctival and corneal xerosis tend to occur after long periods of deficiency [8].

Approximately 85–90% of patients with CF suffer from pancreatic insufficiency, which predisposes them to a reduced enterohepatic circulation of bile acids leading to malabsorption of fat and fat-soluble vitamins (A, D, E, K), and reduced levels of RBP, which is essential for transport of retinol from the liver to tissues [9].

Night blindness in children with CF has been reported since the late 1980s [10]. Those reports drove the interest in liposoluble vitamins with the publication of the largest prospective study on children with CF reporting vitamin A deficiency in 29% of the studied population whereas vitamin E and D deficiencies were detected in 22% and 25%, respectively [11].

More recently, the prevalence of vitamin A deficiency in adolescents with CF has been assessed. A large retrospective study from Australia found an increasing prevalence from 10% in 2007 to 13% in 2010 [12], while a more recent study on a Dutch pediatric population found a prevalence of only 2% [13]. However, those studies do not report any patients with clinical symptoms.

Night blindness has been also attributed to conditions of intestinal malabsorption, in particular in patients who have undergone intestinal bypass or bariatric surgery (even a long time after surgery) [14,15]. Regarding short bowel syndrome in particular, a prospective study on adults demonstrated that subjects undergoing intestinal rehabilitation have a high risk of developing vitamin A deficiency, especially after HPN weaning [16].

Furthermore, the residual intestine can undergo adaptation due to structural and functional changes, leading to an increase in surface area and enhanced nutrient absorptive capacity. This adaptive process is characterized by increased villus height and crypt depth as a result of cellular hyperplasia. Several lines of evidence have implicated vitamin A as a potential modulator of intestinal mucosal adaptive growth in rat models of massive small bowel resection. The speculative mechanism seems to be via retinoid-responsive genes, which were shown to be upregulated in the adapting remnant small intestine at early time points after resection [17,18]. In addition, the same group of researchers demonstrated that VAD seems to inhibit intestinal adaptation after small bowel resection because of crypt cell proliferation reduction, crypt cell apoptosis augmentation and a reduction in enterocyte migration rates [19].

Finally, liver disease could also be a cause of bile acid deficiency and reduced micellar solubilization fat malabsorption, or the inability to synthetize RBP which is needed for the transport of vitamin A [2]. Furthermore, VAD may also exacerbate cholestasis due to possible excessive intrahepatic bile duct proliferation, which has a negative effect on the digestion of lipids [20]. Cholestatic liver disease in our patient is probably multifactorial and mediated by the sum of CF related liver disease [21], which could account for the precocity of liver damage, and the development of intestinal failure-associated liver disease [22] (Figure 1).

Normal findings in the other lipid soluble vitamins (namely D, E, K) demonstrates once more the peculiarity of vitamin A metabolism.

The intestinal absorption of lipid soluble vitamins has been studied in a cellular model and found to be different for vitamin A in comparison with the other lipid soluble vitamins. This model also demonstrated that vitamin A has a competitive absorption pathway with the other three lipid soluble vitamins, which could partially explain the different values found in our patient [23].

Another mechanism for the other lipid soluble vitamins is probably related to the central role of liver absorption and storage, which has been demonstrated to be impaired in patients with chronic liver diseases [24].

In conclusion, this case report nicely illustrates the central issue of vitamin A deficiency and the key factors leading to the “vicious cycle of vitamin A deficiency” [25]. Patients with conditions impairing intestinal absorption associated with liver disease could be at risk of VAD development; thus, it is essential to monitor serum vitamin A level. When these conditions are particularly severe, oral supplementation could be insufficient, and the parenteral route of administration could be required.
